# Lighting-environment-adjustable block-type 3D indoor PV for wireless sensor communication

**DOI:** 10.1038/s41598-023-45226-9

**Published:** 2023-10-19

**Authors:** Yeon Hyang Sim, Jung-Hyun Hwang, Min Ju Yun, Kyoungho Lee, Dong Yoon Lee, Seung I. Cha

**Affiliations:** 1 Energy Conversion Research Center, Electrical Materials Research Division, Korea Electrotechnology Research Institute, 12, Jeongiui-Gil, Seongsan-Gu, Changwon, 51543 Korea; 2 Power SoC Research Center, Power Semiconductor Research Division, Korea Electrotechnology Research Institute, Changwon, Korea; 3 Department of Electrical and Electronic Engineering, Pusan National University, Changwon, Korea

**Keywords:** Devices for energy harvesting, Solar energy

## Abstract

Demand is increasing for photovoltaics (PVs) as a result of the development of the Internet of Things and edge computing technologies. As the lighting environment is different for the applications, thus, PVs must be adjustable to various light environments in which systems are installed. PVs should therefore be capable of easily changing their morphology without damaging the cells. To address this problem, in this work, a three-dimensional (3D) structure that increases power output under omnidirectional light was applied to a crystalline silicon solar cell array using a block-type method. The resultant block-type 3D indoor PV could operate a Bluetooth low-energy module in conjunction with a power management integrated circuit when the illuminance was 532 lx and 1620 lx and each PV installation area was 129.9cm^2^ and 32.48 cm^2^ respectively.

## Introduction

With the recent development of the Internet of Things (IoT)^1,2^ and edge computing^3,4^ technologies, the individual power source for electric devices including wireless communication for transmit sensed data with ZigBee, Bluetooth low energy (BLE), and Bluetooth classic devices are needed for more feasible deployment. The process of signal transmission consumes less than 600 mW in a Bluetooth classic device^5^, less than 100 mW in a ZigBee device^6,7^, and less than 30 mW in a BLE device^7–11^. Therefore, a PV system must generate at least 30 mW of power to operate a BLE device; however, most IoT devices are installed indoors, where light is dim and diffused.

Organic photovoltaics (OPVs)^12–14^, perovskite solar cells (PSCs)^12,15^, and dye-sensitized solar cells (DSSCs)^12,15^ have been considered as indoor PVs (IPVs) because of their high power output under dim light, whereas crystalline silicon (c-Si) solar cells have been excluded from this application because of their low power output under low-light conditions^12^. However, OPVs, PSCs, and DSSCs are not mass-produced and are difficult to scale-up for use in real applications^16–18^. As a result, they are expensive to manufacture and difficult to incorporate into a scaled system. Thus, only commercially available c-Si solar cells should be used to meet immediate needs of real applications. Consequently, a higher power output in the same installed area needs to be achieved even in low-light-intensity environments through morphological modifications so that not only c-Si solar cells but also other PV modules can be used.

**Figure 1:**
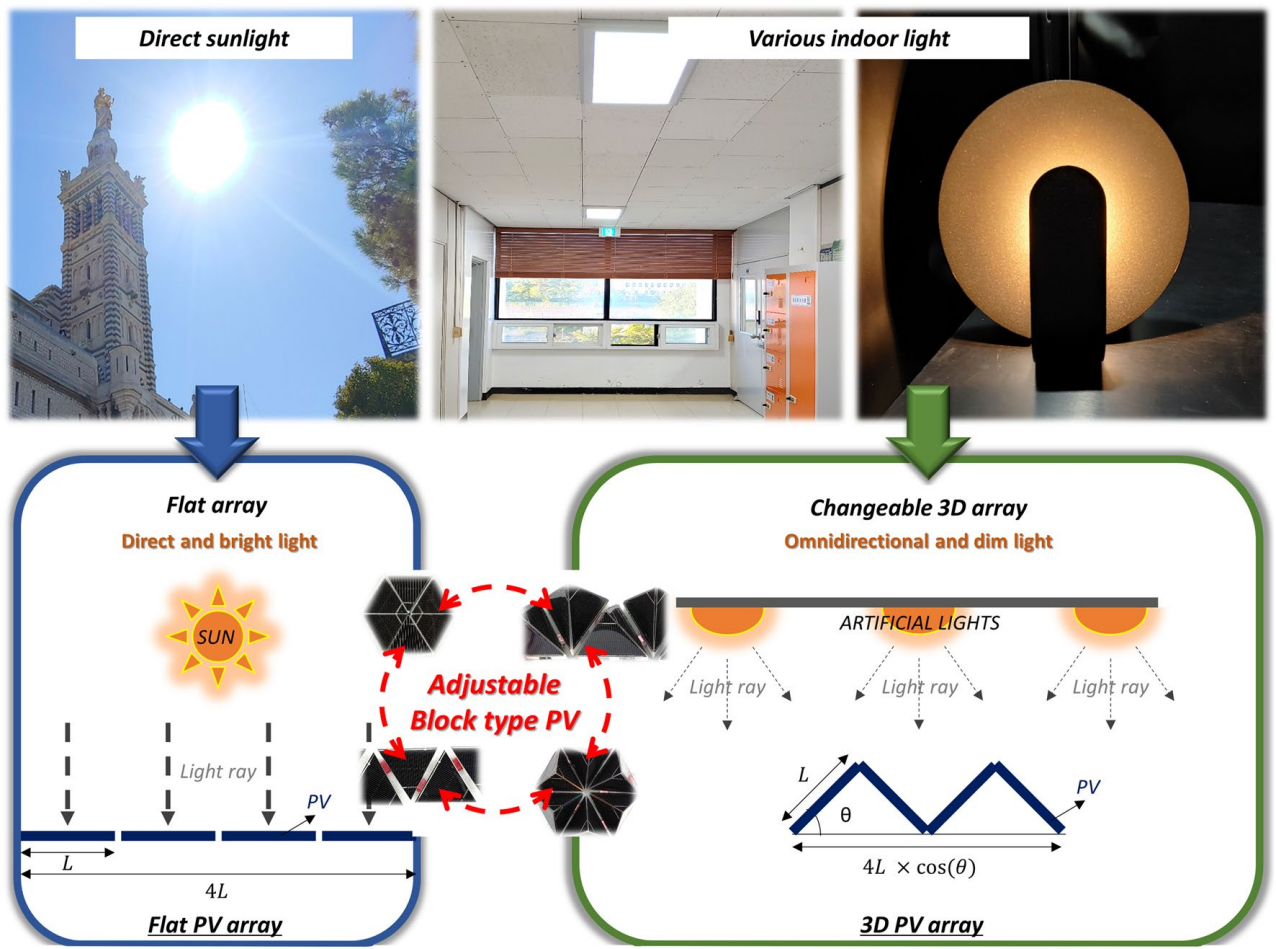
Conceptual image of the adjustable c-Si solar cell array considering different light environments.

In the present paper, given the possible range of indoor light conditions, we propose a method to overcome the low power output of a c-Si solar cell under dim light through a morphological treatment. Unlike sunlight, the light in an indoor environment strongly depends on the user and the usage environment; in addition, the source type varies. Furthermore, under most indoor conditions, the light sources are fixed to the ceiling of a room and are arranged periodically, resulting in diffused rather than direct light. Given the variability in such lighting environments, a flat PV module results in underutilization of the available light. A change in the three-dimensional (3D) configuration of the structure provides the advantage of an angled PV module and leads to the capture of incident light with a greater range of incident angles. A one-size-fits-all approach is difficult in this situation; the few-sizes-fit-all approach is more appropriate to adjust various lighting environments effectively. To satisfy the few-sizes-fit-all method, we have implemented the block-type method as a simple and easy technique to change the morphology of cells; this technique enables a PV array to be assembled as easily as toy blocks. This approach also increases the durability of the array, and the arrangement can be adjusted as shown in Fig. 1 to suit specific lighting environments. As a prototype, tetrahedral and flat structures have been fabricated and their arrangements have been demonstrated to change upon the separation and insertion of PVs in a block-type structure.

In addition, we incorporated a power-management integrated circuit (PMIC) for stable processing and communication with a BLE sensor module. We have demonstrated that 3D c-Si unit array could operate a humidity and temperature sensor without additional power input and the operation was successful at 535 lx with a 129.90 cm^2^ 3D c-Si unit array and at 1620 lx with 32.48 cm^2^.

Although the c-Si solar cell was used in this research, we could successfully operate the BLE module using block-type c-Si solar cell array less than 200 cm^2^ under ~ 600 lx and less than 100 cm^2^ under ~ 1700 lx. It could be possible by adopting the few-sizes-fits-all method and demonstrating the PMIC. These methods are also applicable to other low-light high-efficiency PV systems, and it is expected that better results will be obtained.

## Results and discussion

To extend the applications of PVs, changing the target systems from large-scale solar plants to urban PVs is critical. In particular, IoT devices and edge computing systems require wireless power sources, and batteries that require charging after discharge are currently used in these applications. Therefore, self-powered devices such as PVs are required. Among various available PV systems, c-Si solar cells, which can be mass produced and exhibit good long-term stability, were used in the present study. However, because most of IoT and edge computing systems are installed in indoor environments, using PV systems under low-intensity light necessitates an evaluation of the optimal PV morphology for indoor lighting conditions. In addition, because the numerical light distribution depends on the light source, we need to determine which structure is preferred for each light condition. In our previous study, 3D structural c-Si solar units were found to be useful for indoor, highly diffused light because of their increased surface area compared with that of a flat c-Si unit with the same installed area (Fig. 1). A 3D-structured PV needs to be adjustable to operate under various light conditions. A few PV designs enable the fabrication of PV array arrangements and PV cell structures that are changeable. However, because of the fragile characteristic of c-Si solar cells, the devices cannot be stretched or flexed without additional treatment^19,20^. Therefore, we adopted a few-sizes-fit-all approach to use c-Si cells under various light conditions.

### Adjustable PV array

**Figure 2:**
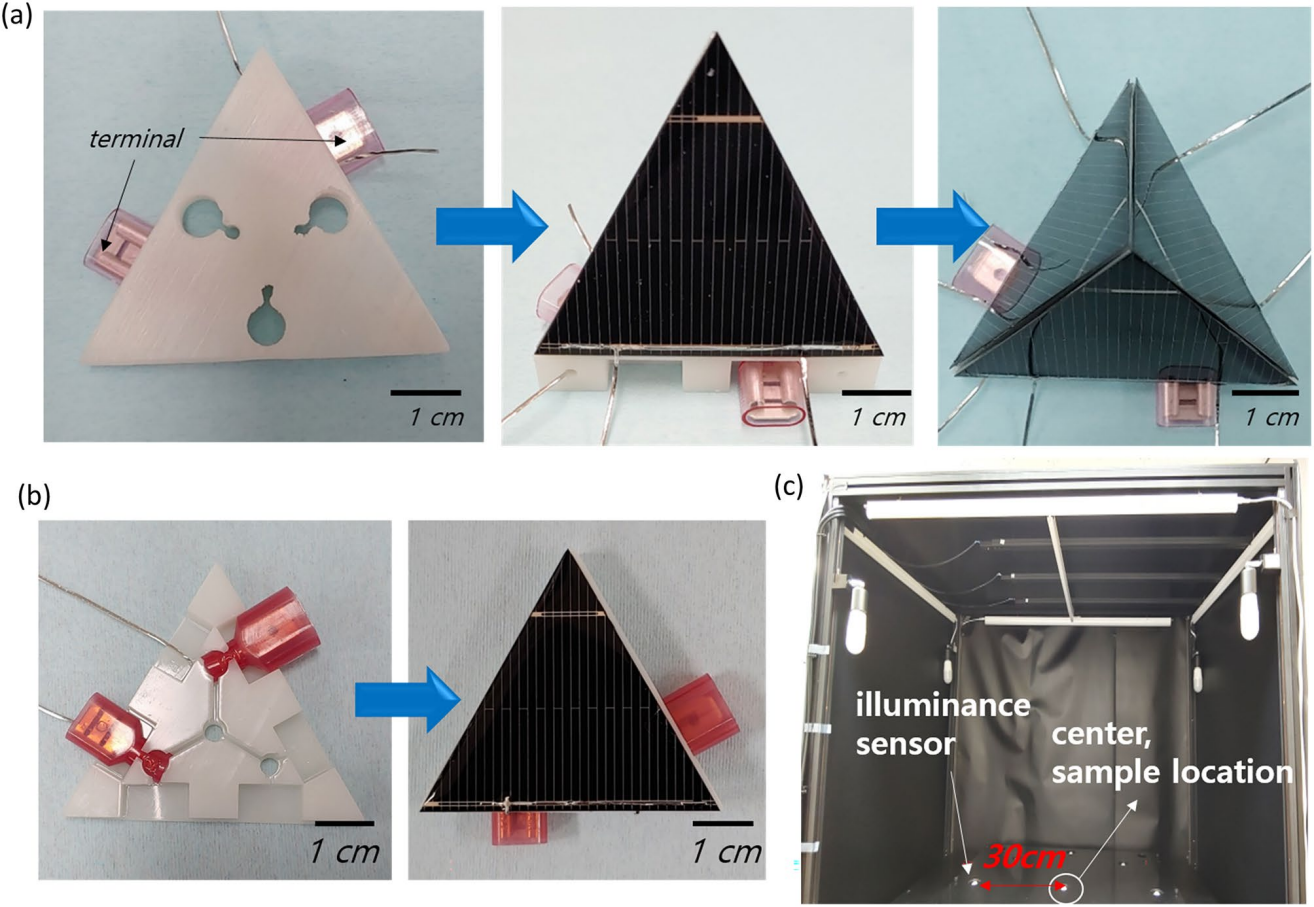
Manufacturing process of a block-type module for (**a**) a 3D unit and (**b**) a flat cell. (**c**) Image of the measurement box.

For a c-Si solar cell to be used as an IPV, it should exhibit a change in morphology in response to various light conditions. First, robust components such as a metal frame, window glass, and back sheet, which prevent structural changes of a conventional PV module, were removed from a PV module to enable changes in morphology. As substitute materials, rear side frames were incorporated into the c-Si solar cell to provide mechanical strength, and silicone encapsulation was used at the front side as a substitute for the window glass of a conventional module. Specifically, we used block-type frames as a few-sizes-fit-all module that enables easy assembly among units. As shown in Fig. 2a, the cells with block-type frames were assembled by insertion at the bottom frame, similar to a toy block. With this method, which we refer to as the force-separation method, the mechanical force components and electrical connection lines could be separated. In the 3D structure, the electrical connection joint is the main component subjected to force and it gradually cracks or breaks away from the cell, causing electrical degradation. Therefore, only the terminals were subjected to force during assembly and disassembly. The terminal at the bottom frame satisfied both concepts (i.e., the block-type method and the force-separation method at the electrical connections). In addition, the angle of the 3D structure was found to be well maintained and reproducible. A flat cell was also constructed as a block-type structure (Fig. 2b) but was directly attached at the bottom frame.

### 3D unit for indoor photovoltaics

The adjustable block-type array was successfully connected while maintaining its shape in both the flat and 3D structures. For an accurate comparison, equilateral-triangle-shaped c-Si solar cells were used for both the flat and 3D structures, which measured 5 cm on each side. For the basic comparison, the PV characteristics of c-Si solar cells in flat and 3D c-Si solar cells were measured under 1 sun (1.5 AM) conditions. In both cases, eight samples were measured; as shown in Table 1, the average power conversion efficiency of the 3D unit was 22.8% and that of flat cells was 23.2%, which is only a 0.4% difference. As shown in Table 1, the average short-circuit current density (J_sc_) of the 3D unit mainly affected the decrease in average power conversion efficiency, but because the 3D unit had a higher fill factor, it maintained the average power conversion efficiency similar to flat. The average open circuit voltage (V_oc_) was slightly lower than flat in 3D unit but it did not significantly affected the average power conversion efficiency. In addition, images of the measured samples (see Figure S1a,c) and current–density curves with power-density curves according to the applied voltage are presented in Figure S1b,d. The energy conversion efficiency was as high as 24.1% (flat) and 23.8% (3D), which means that the energy losses of the 3D unit did not strongly affect the PV characteristics because of the force separation, suggesting that further testing under artificial light should be sufficiently reliable. Both the flat and 3D units were then successfully connected as shown in Figure S1e, enabling the arrangement to be changed between straight and hexagonal types.

**Table 1:** PV characteristics of the flat cell and 3D unit under 1 sun (1.5 AM) conditions. The reported values represent the average value of eight cells or units.

Type	Efficiency (%) (SD)	*V*_oc_ (V) (SD)	*J*_sc_ (mA/cm^2^) (SD)	Fill factor (SD)
Flat cell	23.210 (0.761)	0.668 (0.004)	56.195 (0.872)	0.619 (0.024)
3D unit	22.762 (0.626)	1.900 (0.011)	16.467 (0.643)	0.728 (0.017)

**Figure 3:**
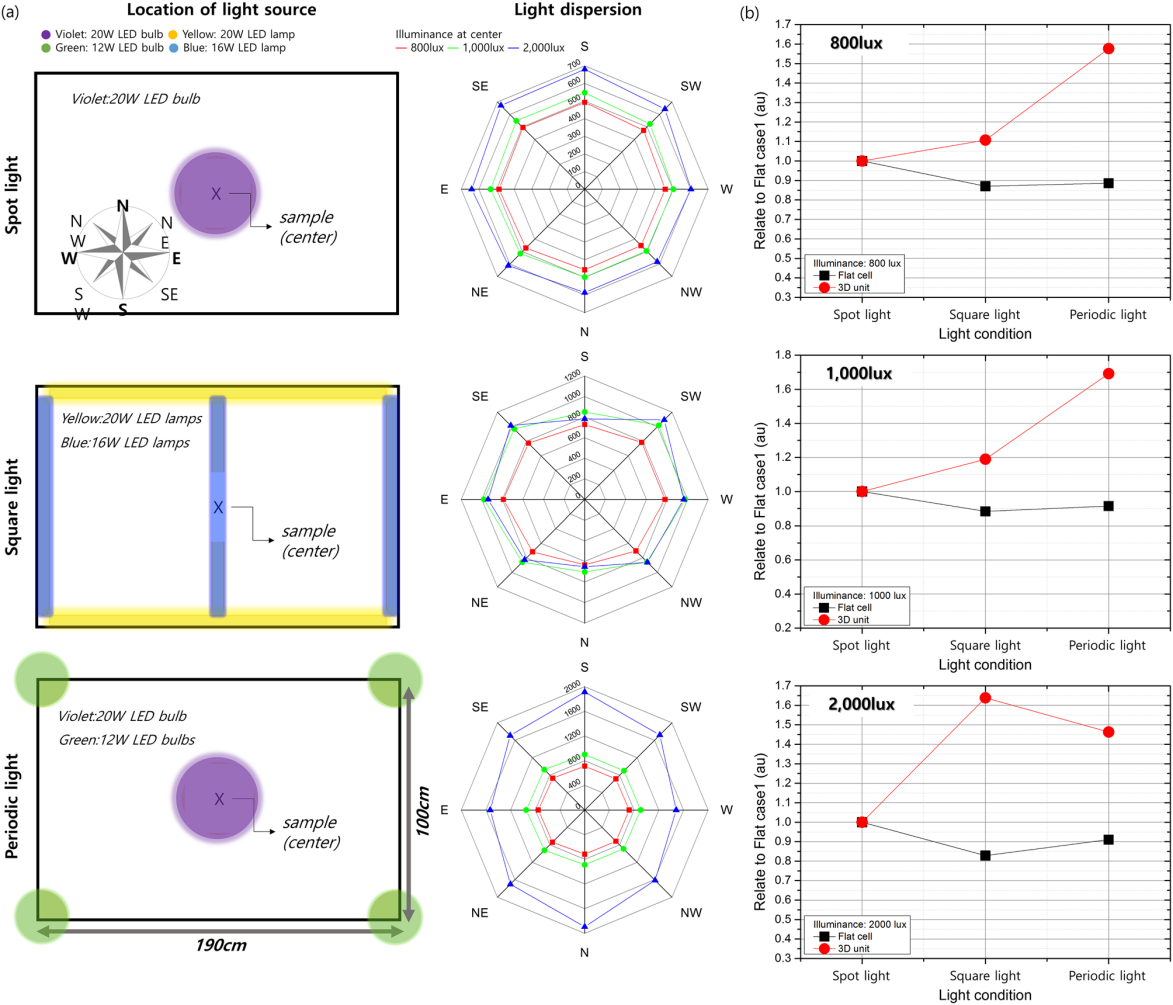
(**a**) Schematics of the location of the light sources for the three conditions: spot light, square light, and periodic light (left), and the light intensity at eight points 30 cm from the center, according to the light intensity at the center (right). (**b**) The power output relative to the spot light condition at 800, 1000, and 2000 lx with a flat cell and a 3D unit.

For the analysis under light-emitting diode (LED) light, we used blackout curtain to block light from outside and then applied a black sheet to the aluminum frames (measurement box) so that only the light inside the measurement box illuminated the devices. The light intensity was measured at the bottom of the measurement box (Fig. 2c), and the light source could be moved up and down with a lift machine to vary the light intensity. Therefore, three different light conditions were used in the experiments (Fig. 3a). Specifically, we tested three different light intensities—800, 1000, and 2000 lx—because the light intensity in our offices and lab was ~ 800 lx at a work desk but was greater than 1600 lx at the top of a shelf.

First, a particular spot was illuminated by only one light source, referred to as the spot light condition; the location of the light source is shown in Fig. 3a. The results in Fig. 3a show that the intensity of the light in the area illuminated under the spot light condition was uniform if the distance was the same in all directions; the difference in light intensity between the center and the periphery (30 cm from the center) indicated that the spot light condition was the most directive light condition. Because the spot light condition had less angled light, the flat cell exhibited greater power output than the 3D unit at all of the investigated intensities (Table 2 and Figure S2). Since c-Si solar cells mainly generate electric power under sunlight, which is direct light source, research has been conducted to optimize solar cells for direct light^21,22^, and as a result, differences in power output were confirmed under spot light condition. Therefore, the angle of incident light of flat cell was mostly 0°, which is the optimal condition for commercialized solar cells, whereas the angle of incident light of 3D unit was close to 70°, the power output of the 3D unit decreased. By contrast, the light dispersion under the square light condition was largest among the three investigated conditions because of the tube-type light sources (Fig. 3a) but was still uniform at the same distance from the center except under 2000 lx. Thus, the power output of the 3D unit was slightly greater than that of the flat c-Si solar cell because of the increase in intensity of diffused light. Under the periodic light condition, which was the least uniform light environment among those investigated, five light sources were used—the same as under the square light condition—but were bulb-type sources (Fig. 3a). Thus, the light intensity and angle both varied under the periodic light condition. Similar to the square light condition, this more omnidirectional condition led to a greater power output by the 3D unit compared with that of the flat cell. Notably, even though the light intensity at the center (where the samples were located) was the same under the three investigated light conditions, the power output differed dramatically (Fig. 3b). Compared with the power output under the spot light condition, that under the dispersive conditions (square light and periodic light) was lower in the 3D unit. By contrast, the power output under the spot light condition was greater under the dispersive conditions in the flat-cell unit. At 2000 lx under the spot light condition, the flat c-Si solar cell showed 40% greater power output than the 3D unit; by contrast, the power output of the 3D unit at 1000 lx under the periodic light condition was 45% greater than that of the flat cell because the spot light and periodic light conditions were the most directive and diffused conditions, respectively. These results show that the PV structure should be changed according to the light environment; in particular, changing the PV structure is critical for indoor light because of the variations among light environments.

**Table 2:** Power output with the flat cell and the 3D unit under LED light. The light intensity was measured at the same location on the sample.

Light intensity (lux)	Type	Power output (μW) (SD)		
		Spot light	Square light	Periodic light
800	Flat cell	174.06 (17.71)	151.54 (15.05)	154.22 (16.05)
	3D unit	138.28 (7.85)	153.12 (4.55)	218.08 (9.89)
1000	Flat cell	240.80 (19.09)	212.76 (18.75)	220.22 (19.87)
	3D unit	189.98 (8.12)	226.00 (7.16)	321.41 (18.66)
2000	Flat cell	630.42 (27.55)	522.27 (30.74)	574.06 (29.81)
	3D unit	451.28 (16.19)	739.60 (21.16)	660.38 (42.75)

### Applying a power management integrated circuit

We attempted to operate an electric device that contained a humidity and temperature sensor with a BLE communication module, which is commonly used in actual applications. The sensor used in this study could communicate every 4 s, and operate 60 ms per communication. At idle state, the sensor consumed 1.33 mW. During BLE communication, 18 mW ~ 36 mW was consumed depending on the sensor operation settings. However, the power output from the PV was unstable because it was strongly dependent on the light environment (e.g., the light intensity and position). Thus, the power output must be managed stably to operate a BLE module. In the present paper, we adopted a PMIC with no additional power input; that is, the PMIC operates only with input energy from the PV. The voltage output of the PV was too low (~ 600 mV) to operate the BLE module, and the PMIC was therefore designed to boost the output voltage of the PV sixfold and then charge the energy storage device (a capacitor in this case) until the charged voltage was sufficient to operate the BLE module. Because charging a storage device with the load (BLE module) connected is difficult, the load connection control circuit disconnected the load until the energy storage voltage reached 3.5 V. It then connected to the BLE module, and when the BLE module consumed power and the storage voltage decreased to 3.0 V, it disconnected from the BLE module and charged the storage device again. To demonstrate the actual application of the self-powered BLE module with a PV module, we performed a test on top of a shelf in our lab. Sixteen LED lamps were installed at the ceiling in periodic fashion, and the shelf was subjected to diffused light conditions, which are commonly encountered in interior spaces. Even on the same shelf, the light intensity differed because of the locations of the light sources (Figure S3). We chose two locations for the test: Shelf 1, which was farther from the light sources, and Shelf 2, which was closer to the light sources at the top of the shelf. At Shelf 1 and Shelf 2, the illuminance during the test was 535 and 1620 lx, respectively. Before the test was started, the flat cell was adjusted to have an output voltage similar to that of 3D unit to ensure matching with the PMIC and the BLE module; thus, the flat unit was constructed with three flat cells connected in series.

**Figure 4:**
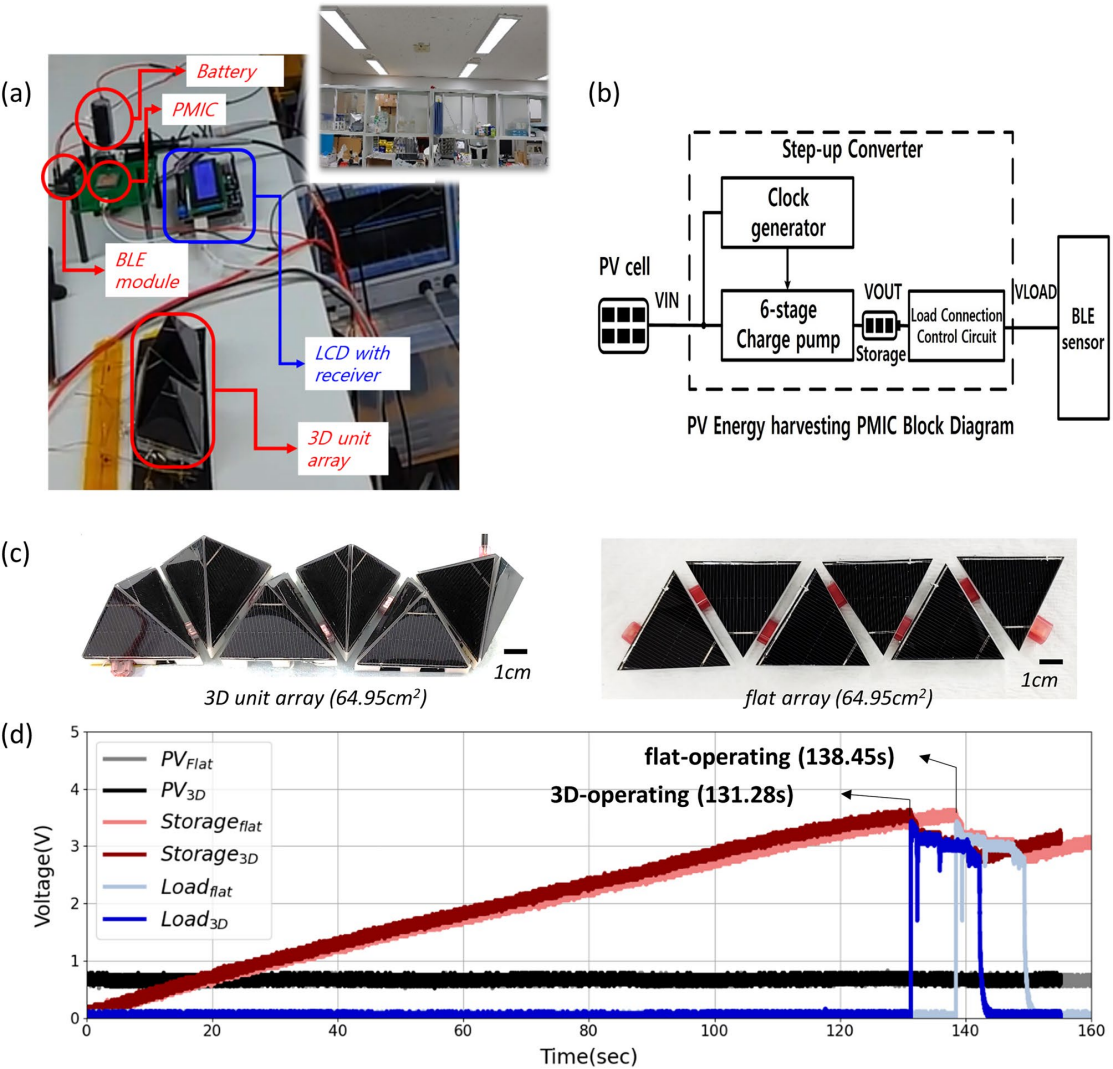
(**a**) Image of the setup on a shelf in our lab; the inset image shows the light condition. The liquid crystal display (LCD) with a receiver and oscilloscope in (**a**) have an external power input. (**b**) Block diagram of the PV energy-harvesting PMIC. (**c**) Images of the adjustable array. The voltage of the PV, storage, and load with fully discharged storage with flat and 3D array (**d**) of 129.90 cm^2^ at Shelf 1 (535 lx).

For testing, the 3D unit array and PMIC circuit with a BLE module was set up as shown in Fig. 4a. Also, the diagram of the PMIC is presented in Fig. 4b. Figure 4b shows a block diagram of the architecture of the PV energy-harvesting system designed in the present study. The input power harvested from the PV cell is provided to a clock generator and a charge pump^23,24^. The clock generator generates clock to be fed to each charge pump for voltage boosting^25^. The charge pump has six stages, and the voltage equal to the clock is boosted at each stage. Because the voltage of the clock is equal to the input voltage, the output voltage is six times the input voltage. The output voltage of the charge pump is used to charge the energy storage device; however, the load connection control block disconnects the sensor (load) from the energy storage device when charging the energy storage device. The load connection control block disconnects the sensor until the voltage of the energy storage reaches a set voltage and then supplies power to the sensor when the voltage of the energy storage device exceeds the set voltage sufficient to drive the sensor. Without the load connection control circuit, the sensor draws current from the energy storage device continuously, which makes the energy storage device difficult to charge.

The number of units used in the evaluation of the PV for operating the PMIC was controlled via the block-type method; samples are presented in Fig. 4c. As mentioned, we designed the system to operate the PMIC without additional power input, where the PV system is the only power source; thus, it was necessary to evaluate whether the BLE module could be operated by charging a fully discharged storage device (we refer to this scenario as a "cold start"). In the case of the Shelf 1 condition, the minimum installation area for both the flat array and 3D array was 129.90 cm^2^; however, as shown in Fig. 4(d), the BLE start time was about 7 s shorter with the 3D array than with the flat array. Given that Shelf 1 was a very dim light environment (< 600 lx), the shorter start time shows that the 3D structure generates electricity more than the flat array (about 5%). In addition, this result implies that the PV structure must be deformed from flat to 3D under highly diffused light conditions.

**Figure 5:**
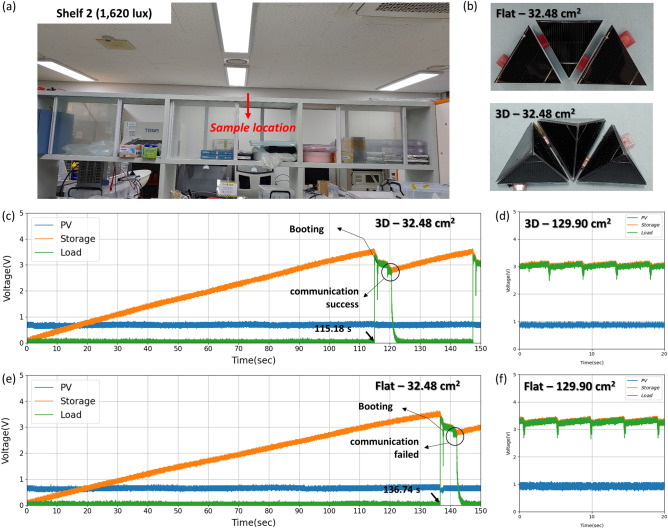
(**a**) The light condition at Shelf 2 (1620 lx). (**b**) The flat and 3D arrays in the same foot area of 64.95 cm^2^. The voltage according to time at Shelf 1 with (**c**, **d**) the 3D array and (**e**, **f**) the flat array. The installation area was 32.48 cm^2^ in (**c**) and (**e**) and 129.90 cm^2^ in (**d**) and (**f**).

Both arrays were also tested under the Shelf 2 condition (Fig. 5a), where the minimum installation areas to start operating the BLE with the flat array and the 3D array were 32.48 and 64.95 cm^2^, respectively, because there was less direct light than below the light source; sample images are shown in Fig. 5b. A video of the arrays operating is available as supplementary information. The difference in cold start performance indicates a difference in the input power of the PMIC from the PV, which affects the operating time of the BLE module and the charging time of the storage device until the setpoint voltage is reached (Fig. 5c,e) even when the installed area of the 3D and flat arrays is the same. The electricity of the 3D array is larger than flat array if installation area was 32.48cm^2^ about 19% at Shelf 2 condition, thus, 3D array succeeded in operating BLE module, while flat array failed it. In Fig. 4c, the electricity of the 3D array is enough to booting and operating the BLE module. In case of flat array, it was booting the BLE module because the voltage of the storage reached to setpoint voltage, but the electricity from flat array is not enough to operating the BLE module. Since the voltage of storage has reached the setpoint voltage, the BLE module was connected to the storage, in order to operate BLE module, the storage should be charged beyond the energy consumption of the BLE module. However, the energy consumption of the BLE module was more than charging the storage with the flat array, it failed to operate. By using a large-capacity energy storage, even if the amount of power generation is small, it could be overcome. If the PV supplies sufficient power to the PMIC, this difference can be neglected because continuous operation is possible (Fig. 5d,f).

In summary, even if the light intensity is the same, if the environment of the light source is different, the PV system also needs to be adjusted into a different form. Therefore, in the present study, a few-sizes-fit-all method was introduced via the block-type method, which enabled operation of a BLE module using an optimized PMIC system.

## Experimental details

### Cell preparation

A stereolithography-type 3D printer (PhotoMonoX, ANYCUBIC) was used to prepare block-type frames using an ultraviolet (UV)-curable resin (UV Tough Resin, ANYCUBIC). The printed frames were cured in a curing machine (Wash & Cure Plus, ANYCUBIC).

Passivated emitter rear cells (LWM5BB; Lightway Solar) were fabricated using a laser. First, the metal ribbons were soldered as electrodes onto c-Si solar cells. The front side of the c-Si solar cells was then coated with silicone rubber (Solaris, SMOOTH-ON) and cured at 70℃ in a conventional oven in air for 30 min. For both the flat and 3D cells, the front side of the cells was placed face down and the frame was placed on the rear side of the cell and subsequently aligned. A liquid-type epoxy (EpoxAcase690, SMOOTH-ON) was mixed in an A:B = 1:1 ratio by weight and then injected through a hole in the frames. The liquid-type epoxy was cured under ambient conditions for 20 h. A terminal was applied to the end of the ribbon of the flat cells in this step.

The 3D cells with a block-type frame were inserted at the bottom frame, and the block-type frame was soldered at the bottom side of the bottom frame (Figure S1). The terminal with metal ribbons was already inserted through the bottom frame and the other side of the ribbons was soldered into the inserted 3D cells to construct a 3D unit. To construct a flat unit, three of the flat c-Si solar cells were directly soldered to each other. The units were connected in parallel via assembly through the terminal.

### Photovoltaic characteristics

The photovoltaic performance of the c-Si solar cells was evaluated under 1.5 AM, 1 sun conditions using a solar simulator (Sun 2000 1000 W xenon source, Abet Technologies) and a source meter (2440, Keithley). The instrument was calibrated using a KG-3 filter and a National Renewable Energy Laboratory (NREL)-certified reference cell.

### LED measurements

A light meter (T-10A illuminance meter, Konica Minolta) was used to measure light intensity, and a source meter (2450, Keithley) was used to measure PV characteristics under LED light conditions.

For the spot light condition, a 20 W LED bulb was installed at the center of the top side of the measurement box. For the square light condition, three 16 W and two 20 W LED tube-type lamps were used. For the periodic light condition, four 12 W LED bulbs were added to the spot light configuration.

### PMIC

**Figure 6:**
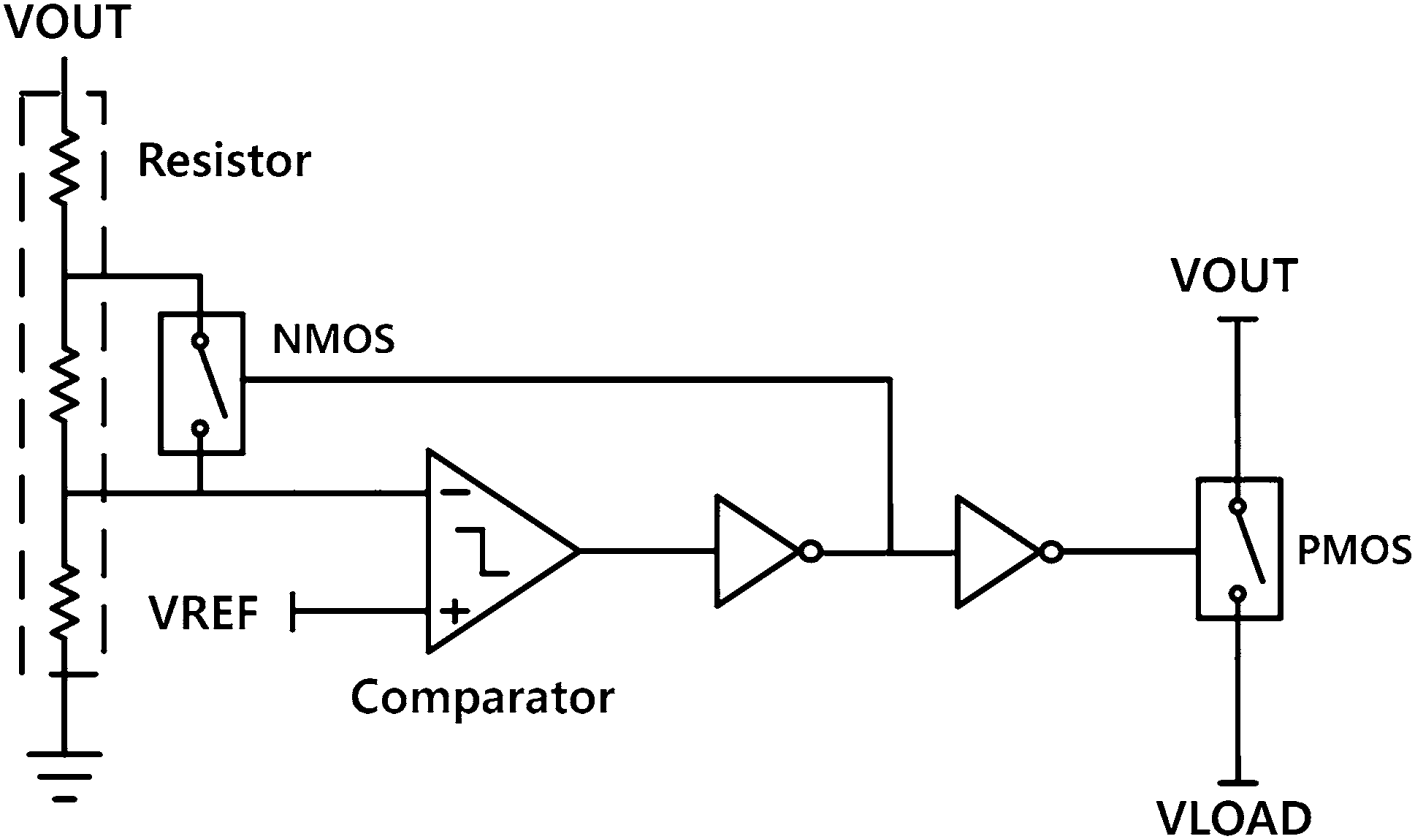
The load connection control block.

We designed a PMIC for indoor-light energy harvesting. The PMIC consisted of a step-up converter and a load connection control block (Fig. 6). Typically, the output voltage achieved through indoor-light energy harvesting is too low to operate an IoT sensor system, requiring a step-up converter. In addition, in the case of a power supply based on energy harvesting, increasing the output voltage while a load is connected is difficult. To solve this problem, we designed a load connection control circuit. Our PMIC was fabricated using a commercial 0.18 μm BCD process.

The power obtained from energy harvesters is typically low-level power. A circuit that uses a source such as an energy harvester has difficultly operating a sensor. Thus, a load connection control block was added to disconnect the sensor during charging of the storage device. As shown in Fig. 6, the load connection control block consists of metal-oxide semiconductor field-effect transistor switches, some resistors, and a comparator. The comparator compares the reference voltage from a beta-multiplier with the output voltage divided by some resistors for connecting the sensor when the output voltage is higher than the set voltage. The set voltage connecting the load can be set by changing the ratio of the resistors. When the voltage of the energy storage device reaches the set voltage, the intermediate resistor is omitted and the ratio is therefore changed. The time to drive the sensor is then secured by hysteresis matching the omitted resistance. In the simulation result, when the input voltage of the PMIC was 600 mV, an output voltage of 1.76 V was obtained without the load connection control circuit, whereas an output of 3.3 V was obtained when the load connection control circuit was used.

## Conclusions

Unlike the direct sunlight, most application environments have dim and diffused light conditions. In addition, light environments differ from one to another. We therefore suggested a few-sizes-fit-all method based on an adjustable PV module. By separating the physical force from the electrical connection and increasing the mechanical strength via the block-type method, an adjustable PV module could be implemented. As a basic transformation, a c-Si solar cell was transformed into a 3D structure for omnidirectional light conditions and the power output of a flat c-Si solar cell under various LED light conditions was increased by 46%. Eventually, with an adjustable c-Si module, the operation of a BLE module with a temperature and humidity sensor was achieved using a PMIC. Under 1620 lx, the BLE was operated by a 3D unit with an area of 32.48 cm^2^, which was one-half the area required for a flat module. Even when a discharged storage device was used, the BLE began operating about 5% sooner at 535 lx than the BLE with a flat array. This result suggests that morphological transformation depending on the light environment is critical. This work represents just the first step toward the practical application of this strategy. In the next step, we have focused on overcoming the limitations of the harvesting module under uneven light using a luminescent solar concentrator, and plans to apply the block-type module concept to the urban environments to prevent electrical losses due to partial shading. To extend the application of PVs, further optimization of the structures for various light environments is needed.

### Supplementary Information

Supplementary Information 1.

Supplementary Video 1.

## Data Availability

All data generated or analysed during this study are included in this published article and its supplementary information files.
