# Supplementary material for: Lighting-environment-adjustable block-type 3D indoor PV for wireless sensor communication
Source: Sci Rep. 2023 Oct 19;13:17846. doi: 10.1038/s41598-023-45226-9 (PMC10587129; doi:10.1038/s41598-023-45226-9)
Supplement: Supplementary file 1 — Supplementary Information 1. [file 41598_2023_45226_MOESM1_ESM.pdf]

**Support Information**

# Lighting-environment-adjustable Block-type 3D Indoor PV for Wireless Sensor Communication

**Yeon Hyang Sim[1], Jung-Hyun Hwang[2,3], Min Ju Yun[1], Kyoungho Lee[2], Dong Yoon Lee[1], Seung I. Cha[1*]**

1. **Energy Conversion Research Center, Electrical Materials Research Division, Korea Electrotechnology Research Institute**
2. **Power SoC Research Center, Power Semiconductor Research Division, Korea Electrotechnology Research Institute**
3. **Department of Electrical and Electronic Engineering, Pusan National University**

*Correspondence to Dr. Seung I. Cha, Korea Electrotechnology Research Institute, 12, Jeongiui-gil, Seongsan-gu, Changwon, 51543, Korea. E-mail: sicha@keri.re.kr; Tel: +82-55-280-1649

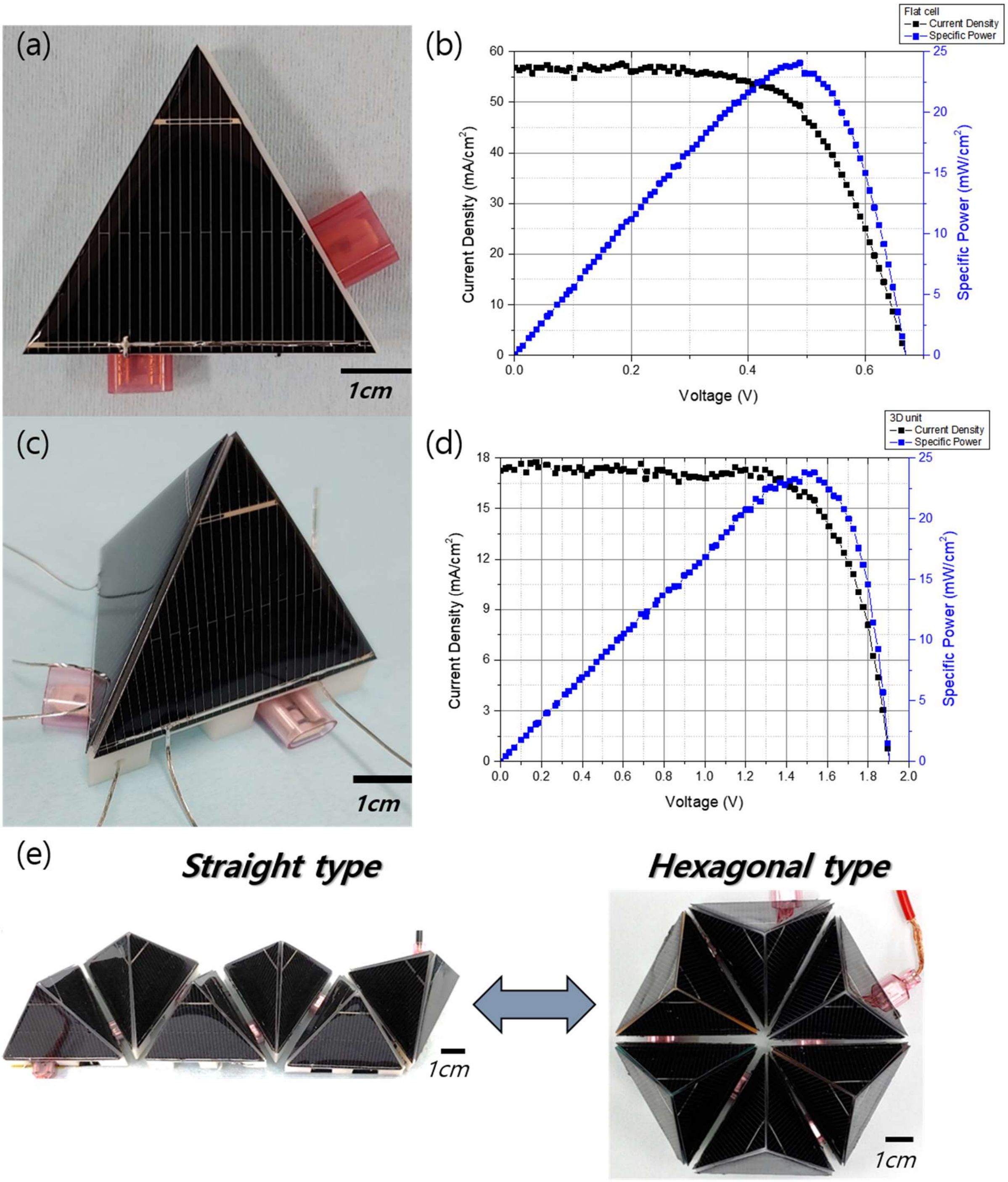


**Figure S1.** A flat block-type c-Si solar cell (a) and its current density and power curve about applied voltage (b). A 3D block-type c-Si unit (c) and its current density and power curve about applied voltage (d). The adjustable PV module which could be transformed into straight and hexagonal structure.

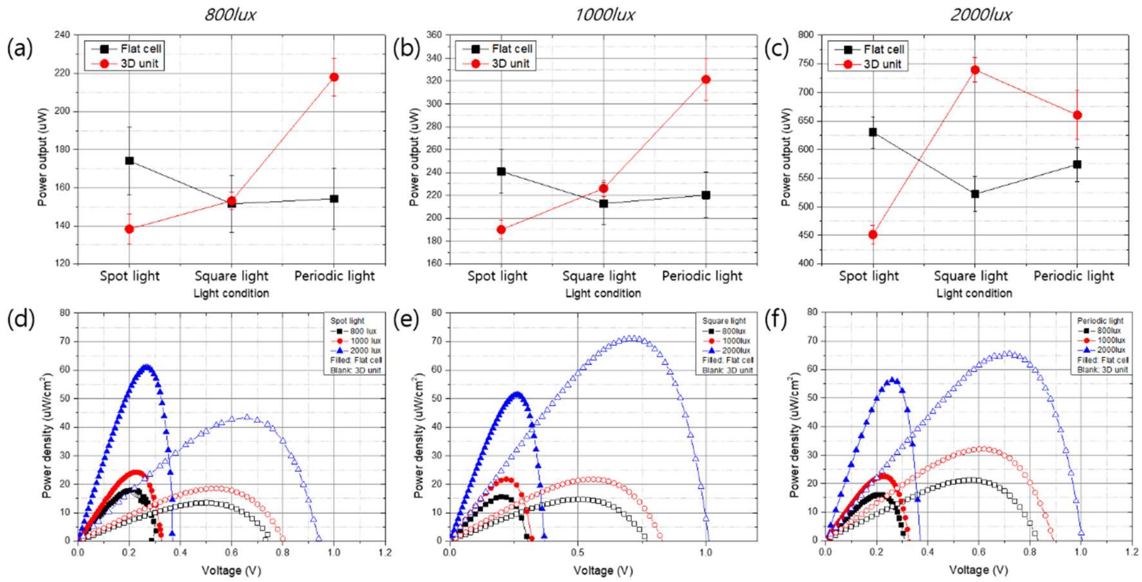


**Figure S2.** The power output of a flat cell and a 3D unit about three different light conditions, under 800 lux (a), 1000lux (b), and 2000lux (c). The power density curve of a flat cell (filled) and a 3D unit (blank) under spot light (d), square light (e), and periodic light (f).

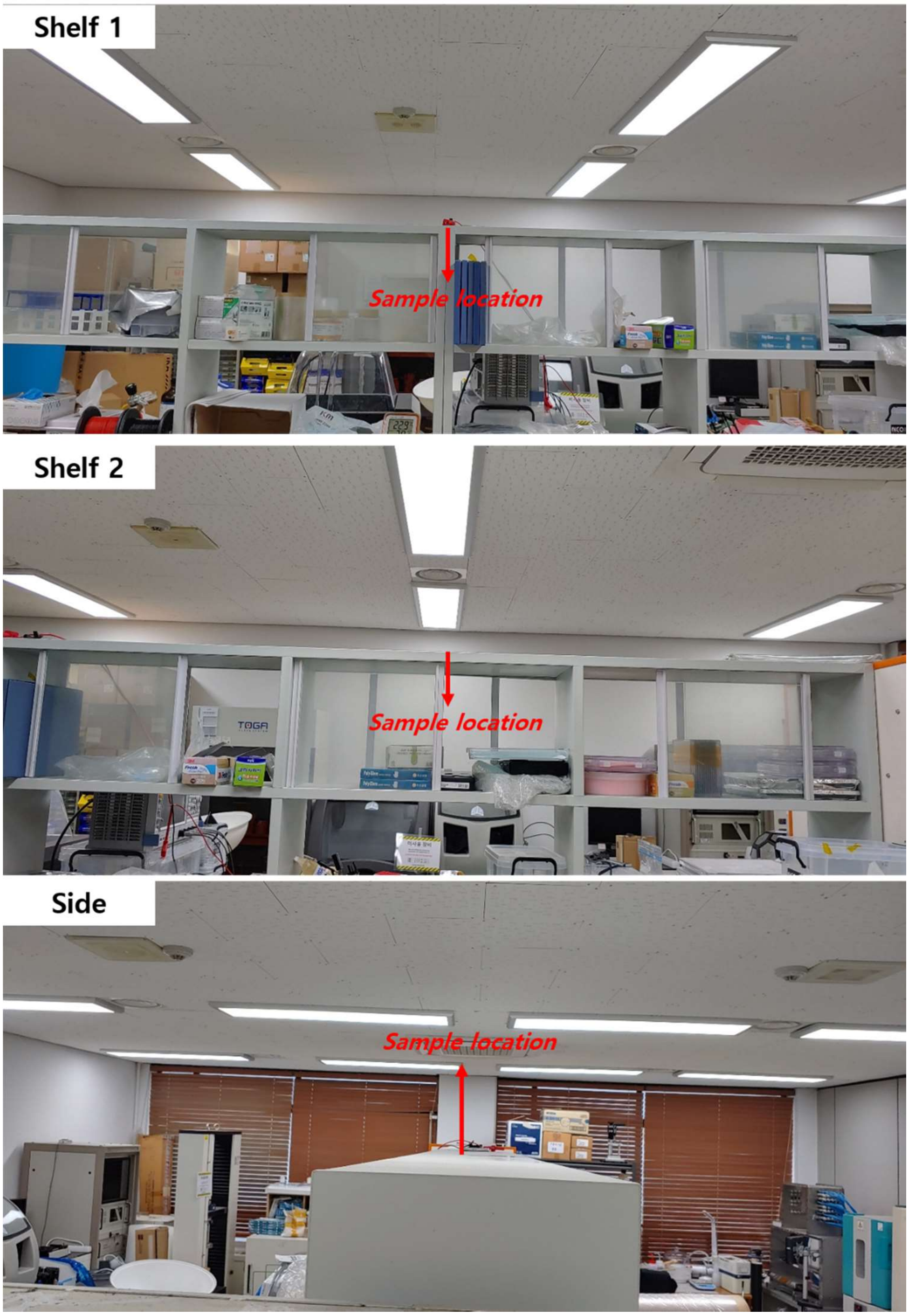


**Figure S3.** The light condition for operating BLE module.
